# Video analysis of 100 matches in male semi-professional football reveals a heading rate of 5.7 headings per field player and match

**DOI:** 10.1186/s13102-022-00521-2

**Published:** 2022-07-16

**Authors:** Johannes Weber, Andreas Ernstberger, Claus Reinsberger, Daniel Popp, Michael Nerlich, Volker Alt, Werner Krutsch

**Affiliations:** 1 Department of Trauma Surgery, University Medical Centre Regensburg, Franz-Josef-Strauß-Allee 11, 93053 Regensburg, Germany; 2 Institute of Sports Medicine, University of Paderborn, Paderborn, Germany

**Keywords:** Heading, Football, Sports medicine, Concussion, Video analysis

## Abstract

**Introduction:**

Heading is an integral part of football and frequent media reports and previous studies about potential danger of heading and head trauma in football fuelled discussions. Epidemiological data and video analyses regarding headings situation and associated head injuries are still missing in male adult professional football.

**Methods:**

In a prospective cohort study in the male fourth German football league, 100 official matches of the 2015–2016 season were assessed by video analysis and a standardized protocol. Heading situations and concomitant circumstances as well as incidents with a propensity of injury (critical incidents) were analyzed. Critical incidents (CI) and seasonal reported head injuries were cross-referenced.

**Results:**

Overall, 11,514 headings were analysed in detail. Video analysis yielded a mean of 5.7 headings per player and match (SD: 1.2; range 0–15). Heading was predominantly performed with the frontal part of the head (76.8%), and nearly two thirds of all headings occurred during defending (65.8%). 71.0% of all headings occured during tacklings, of which 71.9% involved body contact with the opponent player. Video analysis yielded 31 CI on the head due to heading (incidence: 1.02 per 1000 h match exposure and player). 29 CI occurred during heading duels (odds ratio: 5.91), 30 CI with body contact (odds ratio: 28.8) and 6 CI with elbow contact (odds ratio: 6.13).

**Conclusion:**

Heading frequency in male semi-professional football could be determined with a rate of 5.7 headings per match and field player. Cross referencing CI and seasonal reported head injuries revealed a very low number of reported head injuries.

## Introduction

Football is a unique type of ball sports that allows its players to use the head for controlling, passing and shooting a ball [1–3]. For more than 20 years, there has been a growing discussion about the potential harm of headings for the brain [2, 4–9]. This discussion was fueled by the first studies describing several structural and biochemical changes in former football players, even in those without a history of concussion [7, 8, 10, 11], and by the ban on headings for under 13-year old children by the US Soccer Federation (USSF) in 2015. Especially long-term consequences of headings such as neuro-psychological changes are dreaded [10, 12–14]. Epidemiological data on headings and its concomitant circumstances as well as caused injuries by heading situations are sparse, therefore most experiences and evident data on long-term structural changes are derived from other contact sports such as ice hockey or American football. The few interventional studies available on headings mostly describe heading sessions of about 10–15 min [15, 16] with estimated rates of 50 to 100 headings per player. However, it is unclear whether the designs of these interventional studies allow an interpretation of the impact of heading in practial football routine. The present study investigated for the first time heading rates per player and match as well as incidents with a propensity of injury in semi-professional mals by using video analysis.

## Methods

### Study design

In a prospective cohort study, the fourth male German football league, which displays a semi-professional league, were analysed by means of video analysis and injury reports during the 2015–2016 season. Video analysis has been identified as a useful tool for characterising headings in detail and for identifying critical incidents leading to head injuries in football [1, 17]. The investigated fourth male league included 18 teams with professional football players. The Regional Football Association of Bavaria (BFV) provided the television recordings of all 306 matches of the 2015–2016 season for video analysis. The recordings had been filmed by at least one camera fixed to the grandstand of each football stadium. All headings (every head contact or intended or attempted head contact situation with the ball of different players in the match) and incidents with a propensity of injury (critical incident, CI) of the entire season were monitored exploratively by using standardised questionnaires. Seasonal injury reports were obtained by a using a questionnaire and a report handed out to the staff of each of the 18 teams, which allowed an anonymous data collection. Injuries were documented according to previously published injury definitions and data collection standards in football [18, 19].

### Video analysis protocol

**Fig. 1:**
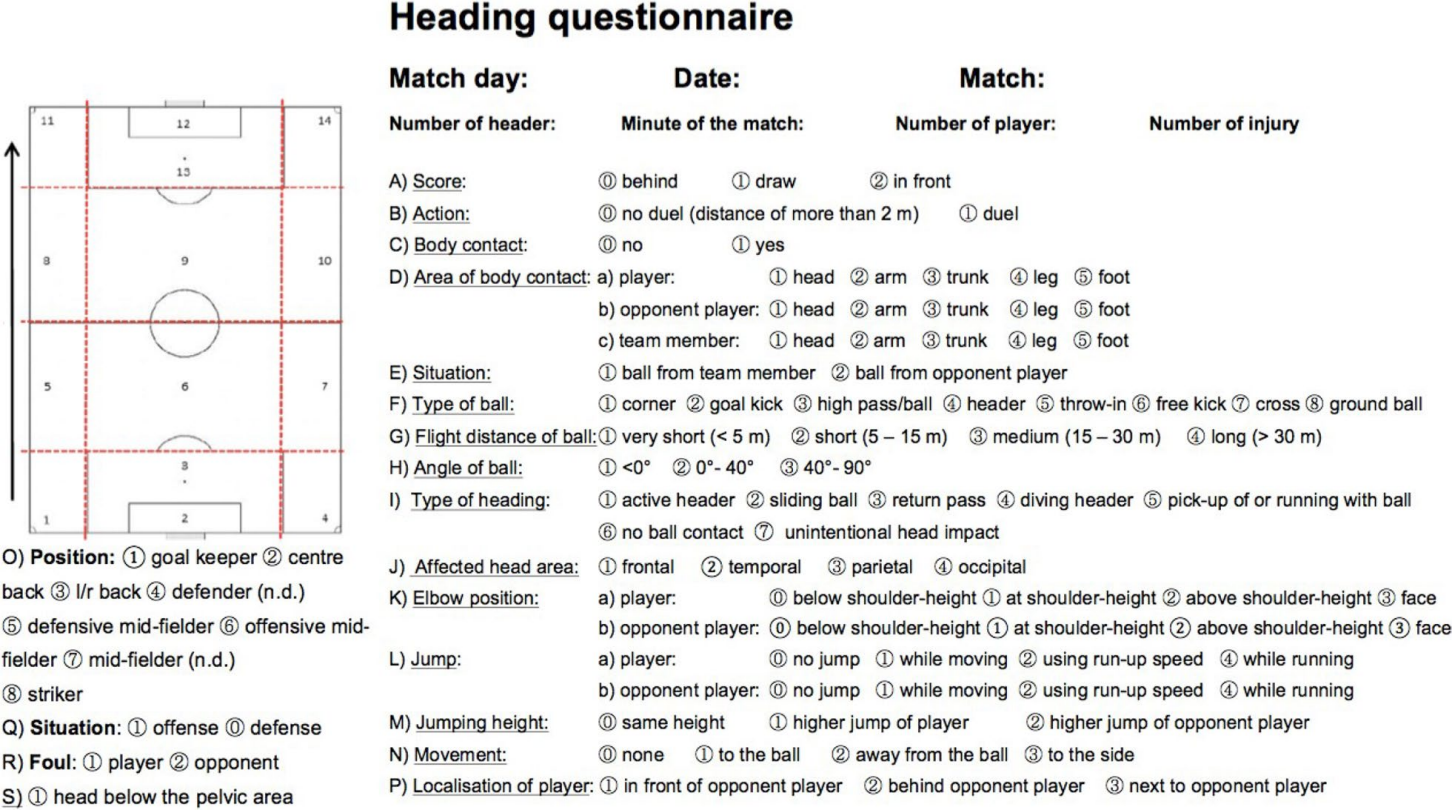
Heading protocol for analysing heading situations in football

A representative, randomly chosen sample of 100 matches was investigated by video analysis. At first, all videos were reviewed to identify the frequency of headings. Every header was then analysed by means of a standardised video analysis protocol developed prior to the study and used here for the first time (see Fig. 1). In this protocol, 18 characteristics of heading were queried including the duelling situation, the areas of body contact or the elbow position during the heading. Furthermore, the type, distance and angle of the ball to the player were documented as well as the impact surface of the ball on the head and any concomittant jumps or movements of the players. Headings were viewed in slow-motion and freeze-frame in a standardised manner to allow a precise examination of the header. Video analysis was performed by three trained raters. Unclear situations were discussed with the video analyzing team online, which consisted of all raters and two supervisors. Because of differences in distances and position of the video camera in each stadium, distances or angles on the video could not precisely be measured. In such cases, auxiliary quantities such as the size and boundaries of the field, penalty areas or centre circles were used to interpret the different heading situations (Fig. 1).

### Critical incident assessment and cross-referencing to reported injuries

Additionally, critical incidents (CI) on the football field according to the criteria first published by Anderson et al. [1] and further developed by Bjorneboe et al. [20] were seperately analysed. According to the previously published standard, a CI is a situation in which the player has a risk for conducting an injury. In the present study, the focus was on risky situations on the head. An incident was recorded if the match was interrupted by the referee and if one of the players involved in the tackling was lying on the ground for more than 15 s or had to be carried off. Every head trauma observed on video was assessed by specific items such as the starting situation and its triggers, the contact area on the head and co-injured body areas. Position on field and evaluation by the referees were also documented. Each CI was viewed several times at different slow-motion speeds and freeze-frames. Any uncertain situation was managed as described above. After match completion, each reported CI was cross-referenced with the standardised epidemiological injury investigation over the season, which was conducted prospectively.

### Statistics

Critical incident and injury incidence were calculated by the number of injuries devided by hours of match exposure multiplied by 1000. Descriptive data such as injury characteristics are presented as absolute numbers and percentages. Rates of contact injuries between different types of heading situations were compared by chi-square test of independence. Odds ratios with corresponding 95% confidence intervals were calculated as effect estimates. A *p* value < 0.05 was considered as statistically significant. All analyses were performed using SAS 9.4 (SAS Institute Inc., Cary, NC, USA).

## Results

### Heading frequency and distribution

**Fig. 2:**
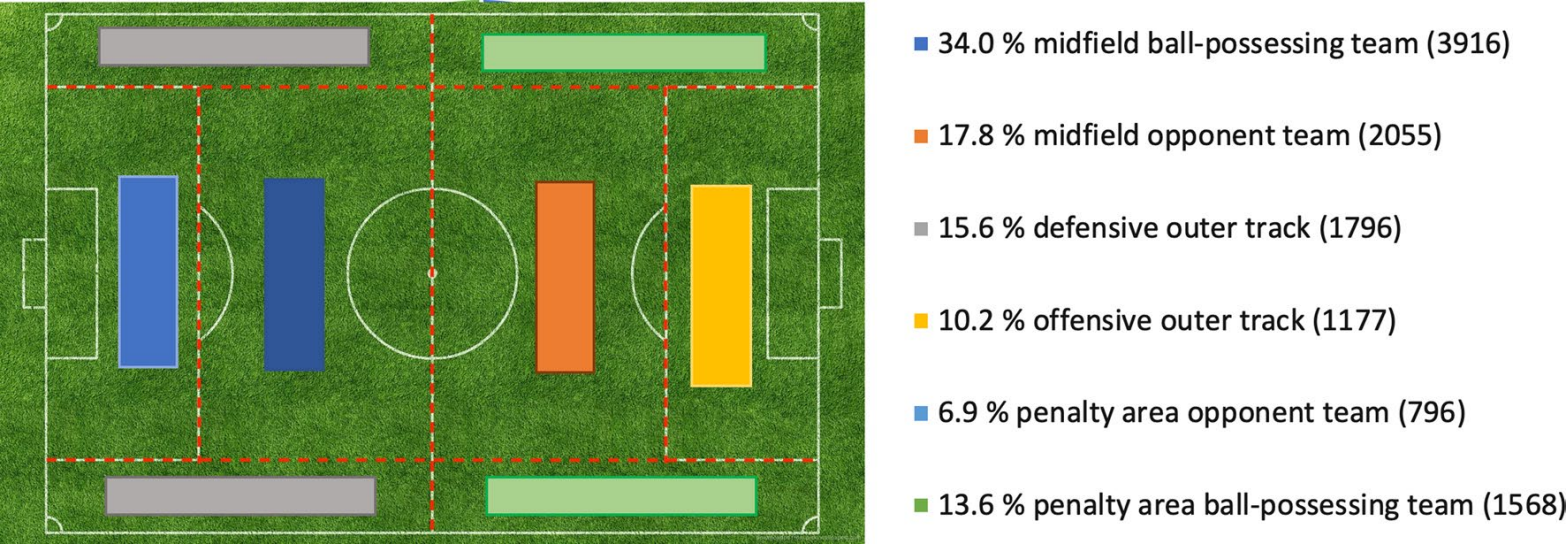
Heading frequency in football field areas

Video analysis yielded 11,514 headings in 9229 match minutes (115.1 headings per match/ standard deviation (SD): 24.4; 1.2 headings per match minute/SD: 0.3, see Table 1). With the exception of goalkeepers, who only performed 7 headings in 100 matches, the mean heading rate per male field player and match was 5.7 (SD: 1.2). While many players had 0 headings in football matches, the highest number of headings per match of one male player was 15. With regard to the position on field, defenders showed the highest percentage of headings (44.1%, 5084 headings) followed by strikers with 12.5% (1435 headings) and midfield players with 35.3% (4019 headings, see Fig. 2). Most headings were performed after high passes (32.0%), goal kicks (18.4%) and previous headings (15.7%). Headings after corners (4.7%), free kicks (8.0%) and crosses (5.0%) were less frequent. In 3.8% (440) of headings the elbow was at or above shoulder-height. The majority of headings was recorded in the midfield area of the football field (5971, 52.8%). The total rate of headings in the penalty area (2364, 20.9%) was lower than in the outer tracks (2973 headings, 26.3%; Fig. 2). About 71% of all headings occurred during tackling, of which 71.9% involved physical contact between the players. Less than 5% of all headings involved body contact with more than one player. About 3% of all headings were judged as a foul by the referee.

**Table 1:** Selected charateristics of the analysed headings in 100 matches

	Total number (percentage)
Position of player	
Defender	5084 (44.1%)
Midfield player	4019 (35.3%)
Striker	1435 (12.5%)
Area on field	
Midfield	5971 (52.8%)
Outer track	2973 (26.3%)
Pentalty area	2364 (20.9%)
Minute of the match	
min 0 to 15	1980 (17.2%)
min 16 to 30	1871 (16.3%)
min 31 to 45	1953 (17.0%)
min 46 to 60	1898 (16.5%)
min 61 to 75	1704 (14.8%)
min 76 to 90	2101 (18.2%)
Playing situation	
Free play	9228 (81.4%)
Free kick	938 (8.3%)
Throw-in	628 (5.5%)
Corner	542 (4.8%)
Type of heading	
Active heading	9402 (81.9%)
Sliding ball	1389 (12.1%)
Pick-up or running with ball	290 (2.5%)
Return pass	115 (1.0%)
Diving header	75 (0.7%)
Unintensional head hit after free kick	62 (0.5%)
Affected head area	
Frontal	8848 (78.5%)
Parietal	2182 (19.4%)
Occipital	163 (1.4%)
Temporal	80 (0.7%)
Elbow position	
Below shoulder-height	11,055 (96.2%)
At shoulder-height	432 (3.7%)
Above shoulder-height	7 (0.1%)
Head position	
At or above pelvic area	11,176 (97.6%)
Below pelvic area	247 (2.4%)
Match score	
Draw	5008 (44.6%)
Own team leading	3161 (28.1%)
Opponent team leading	3067 (27.3)
Ball possession	
Opponent team	7577 (66.2%)
Own team	3865 (33.8%)
Heading duel	
Yes	8178 (71.1%)
No	3324 (28.9%)
Body contact	
Direct contact	5884 (51.2%)
No contact	5616 (48.8%)

### Critical incidents due to heading situations

**Fig. 3:**
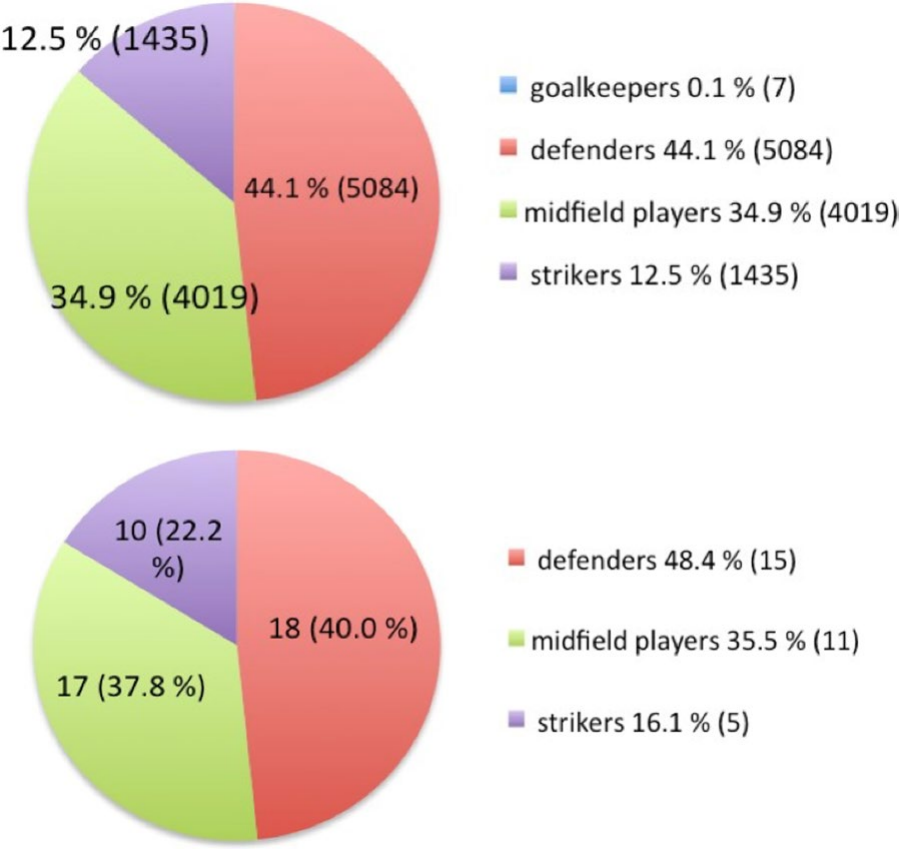
Heading (above) and critical incident frequency (below) in playing positions

Video analysis identified 45 CIs (0.39%) due to heading (see Table 2). The head was the most affected body area (31 players, 68.9%) followed by the back (6 players, 13.3%) and the ankles (4 players, 8.9%). The CI rate for the head per match and player was 0.0016 (1.02 per 1000 h match exposure and player). The distribution of CIs due to heading regarding the playing positions was similar to that of the heading rates with defenders being the most affected players (15 CI, 48.4%; see Fig. 3). 29 CIs (93.5%) involved a time-out of which 5 (16.1%) resulted in the substitution of the injured player. 29 of 31 CIs (93.5%) occurred in duelling situations, and 30 CIs (96.8%) involved active body contact. 13 of 31 CIs (41.9%) were judged as a foul by the referee, and 4 CIs (13.4%) resulted in a yellow or red card. 30 CI (5.1 Cl per 1000 headings) resulted from 5884 (51.2%) headings with body contact, while 1 CI on the head occurred in 5616 (48.8%) headings without body contact (0.18 Cl per 1000 headings; Odds ratio: 28.8; 95%-CI 4.8, 1174; *p* < 0.001). 29 CI (3.5 Cl per 1000 headings) occurred in 8178 duelling situations (71.1%), while 2 CI resulted from 3334 non-dueling situations (28.9%) (0.6 Cl per 1000 headings; Odds ratio: 5.91 95%-CI 1.49, 51.15, *p* = 0.006). 6 CI (13.7 Cl per 1000 headings) were observed in 438 (3.8%) situations with elbow on or over shoulder height occurred, while 25 CI occurred in 11,055 (96.2%) headings without lifted elbow (2.3 Cl per 1000 headings; Odds ratio: 6.13; 95%-CI 2.04, 15.40; *p* < 0.001).

**Table 2:** Selected charateristics of the 31 critical incidents on the head in 100 games

	total number (percentage)
Position of player	
Defender	15 (48.4%)
Midfield player	11 (35.5%)
Striker	5 (16.1%)
Area on field	
Midfield	19 (61.3%)
Outer track	7 (22.6%)
Pentalty area	5 (16.1%)
Minute of the match	
min 0 to 15	0 (0)
min 16 to 30	10 (32.3%)
min 31 to 45	6 (19.3%)
min 46 to 60	2 (6.4%)
min 61 to 75	10 (32.3%)
min 76 to 90	3 (9.7%)
Playing situation	
Free play	28 (90.3%)
Free kick	2 (6.5%)
Corner	1 (3.2%)
Match score	
Draw	11 (36.7%)
Opponent team leading	10 (33.3%)
Own team leading	9 (30.0%)
Basic action of the player	
Air time	28 (90.3%)
Running	2 (6.5%)
Standing	1 (3.2%)
Heading duel	
Yes	29 (93.5%)
No	2 (6.5%)
Body contact	
Direct contact	30 (96.8%)
No contact	1 (3.2%)
Affected head area	
Occipital	10 (32.3%)
Facial	8 (25.8%)
Frontal	8 (25.8%)
Parietal	5 (16.1%)
Direction of attack	
Back	11 (37.9%)
Front	11 (37.9%)
Side	6 (20.7%)
Both sides	1 (3.4%)
Contact to the ground	
No contact with ground	27 (87.1%)
One leg	3 (9.7%)
Two legs	1 (3.2%)
Type of contact	
Collision with opponent player	19 (61.3%)
Hit by opponent player (elbow)	6 (19.4%)
None	3 (9.7%)
Collision with team player	1 (3.2%)
Collision with ball	1 (3.2%)
Hit by team player	1 (3.2%)
Affect on player	
Able to leave the field on both feet	20 (64.5%)
No treatment	9 (29.0%)
Carried off	2 (6.5%)

### Cross-referencing of critical incidents with seasonal injury reports

When cross-referencing the CIs with seasonal injury reports of head injuries, only 4 head injuries had been reported by the teams in the 100 investigated matches. 1 nasal fracture, 1 skin laceration and 2 concussions were diagnosed resulting in a concussion rate after heading of 0.017% and a concussion incidence of 0.065 per 1000 match hours and player.

## Discussion

In the so far largest cohort study on video analysis of heading in male football, this study provides detailled information on the incidence and circumstances of headings in semi-professional football. One important finding of this study was the quantification of heading per player and match with a mean of 5.7 or a maximum of 15 for one player during the match. The impact of heading on neurological or neuropsychological symptoms was previously published in experimental studies with significantly higher heading frequencies per intervention [11, 21–24] than in this study yielding a need for a discussion on how the previous data is applicable to real game situations. The importance of this topic for sports medicine has been documented in several studies over the past few years and the increasing interest in the results of the international conference on concussion in sports. Previously published study results have indicated that heading in football may be dangerous for the brain, changing its microstructure and neurochemistry and effecting neurocognitive changes [7, 8, 10, 11, 25–27]. Additionally, the ban on heading for under 13-year olds by the US Soccer Federation (USSF) in 2015 has given a further impetus to the ongoing discussion about the harmfulness of heading, in particular because scientific evidence on the danger of heading in football is still lacking.

### Epidemiological classification

So far, epidemiological data on heading and head injuries in football are scarce [5, 6]. Detailed video analyses of headings and situations for a propensity for injuries are rare, although such analyses are a commonly used analytical method for characterising specific situations in sports matches [1, 17, 28–33]. Several studies have concluded that traumatic brain injuries due to head trauma, especially when incurred several times within a short period of about 2–4 weeks, are a major risk factor for changes in the brain [34–36]. As this investigation showed, injuries to the head resulting from heading are rather rare in football compared to common injuries predominately affecting thighs, knees and ankle [18, 37, 38]. Head injuries, which also include midfacial lesions and fractures, account for 5–20% of all reported injuries and have been recently considered an underrepresented problem in sports [39]. The total number of sports-related concussion is still often overlooked, in part because of the non-apparent clinical signs that are only revealed by a clinical examination that occasionally medical doctors are not proficient in. Especially at lower skills levels and in junior football there might not even be a medical team available to carry out such an examination [39]. This situation defines the urgent need for improved and sufficent injury prevention steps for head injuries of football players.

### Approach to prevention of head injuries

One other important finding of this study is that CIs on the head and propably head injuries directly result from heading duels, body contacts and high elbow-positioning. Head injuries are known to often occur during tackling, so that different strategies for preventing head injuries have been developed over the past few years [40, 41]. Rule modifications like the advice to ban a player for intentional elbow-to-head contact, have significantly reduced the rate of head injury [1, 3]. However, further strategies should be considered in connection with the results of this study, such as education in fair play in heading duels, avoiding fouls during tackling and training in correct heading techniques [39]. Above all, football players need to be informed about the possible consequences of head injuries to reduce their willigness of risking a contact between their head with the body of other players. Such education of football players may be an important factor for preventing head injuries in heading duels [42, 43].

With a rate of critical incidents and concussions in this study, the risk of sustaining a concussion in football is much lower than in other male sports such as Australian football, American Football or Ice hockey [31, 44]. Nevertheless, the total number of head injuries in male football worldwide is substantial because of the high number of football players, since football is the most important sport worldwide. It is essential to reduce the number of head injuries and to eliminate the uncertainty of football players on this topic. Besides injury prevention of head injuries, both football players and staff should be further educated about the incidence, diagnostics, symptoms and first aid on field [43, 45]. The problem of late diagnosis or overlooked concussion [39] is still a problem in all team sports and should also be improved in football.

One other important result of this study is the low concussion rate of the injury statistics provided by the teams compared to the rate of CIs on the head documented by means of video analysis. According to the definition of a CI, only 2 concussions in 31 CIs to the head were verified in our video analysis. This situation may illustrate that either there is an overcasting for minor head injuries in video analysis or that these minor head impacts are not gaining enough attention of the football players or other staff on field. Potentially overlooked minor head injuries may not be worked up properly (for example by a detailed (neurological) examination) and may not receive sufficent treatment with adequate further clinical diagnostic work up or rest, so that players are at risk of sustaining recurrent hits on the head in further heading duels [45].

### Limitations

This study also has some limitations. At first, all study findings were obtained in male football, so that all observations and conclusions can only be compared or transferred to male sports.

Further on, in the fourth male football league, television recordings were often only obtained by 1 video camera per match and football field. Evaluating heading and situations with a risk for head injuries may therefore be different to football matches in professional football, in which football matches are continuously recorded by several cameras in different positions. Additionally, the transfer of our study results to other football subpopulations is somewhat limited. Professional players may even have better tackling abilities in headings duels than amateur or junior players. The different constitution of football players, especially of women and junior players, influence heading situations as well as the aetiology of injuries. The incidence of heading, CIs and head trauma of football players is generally assessed by match and training exposure, whereas this study only included match exposure. Competitions as well as official matches are assumed to be associated with higher injury rates than training sessions [46].

## Conclusion

The biggest so-far analysis of headings in male semi-proferssional football revealed a mean heading rate of 5.7 per field player and match, which seems to be a lower number than previously estimated in the literature. Analyzation of situations with a propensity for injuries showed that these situations occurred more often after body contact and high elbow positioning, even if cross-referencing with season injury rates only showed low injury and concussion rates. Future research is necessary to investigate heading rates in other subpopulations and, especially, head trauma caused by heading in a larger number.

## Data Availability

The datasets used and analysed during the current study are available from the corresponding author on reasonable request, except insurance data which is property of the insurance itself.

## Abbreviations

BFV: Bayrischer Fußball Verband (Bavarian Football Association)
CI: Critical incident
SD: Standard deviation
USSF: United States Soccer Federation
VBG: Verwaltungsberufsgenossenschaft (General Insurance in Professional Sports)
